# MicroRNAs as clinical tools for diagnosis, prognosis, and therapy in prostate cancer

**DOI:** 10.1016/j.tranon.2022.101613

**Published:** 2023-01-04

**Authors:** Fatima Ghamlouche, Amani Yehya, Yousef Zeid, Hiam Fakhereddine, Jhonny Fawaz, Yen-Nien Liu, Mohamed Al-Sayegh, Wassim Abou-Kheir

**Affiliations:** a Department of Anatomy, Cell Biology, and Physiological Sciences, Faculty of Medicine, American University of Beirut, Beirut 1107-2020, Lebanon; b International Ph.D. Program in Medicine, College of Medicine, Taipei Medical University, Taipei 110, Taiwan; c Biology Division, New York University Abu Dhabi, Abu Dhabi 2460, United Arab Emirates

**Keywords:** Prostate cancer, MicroRNAs, High-throughput profiling technologies, Diagnostic/prognostic biomarkers, Therapeutic tools, PCa, Prostate cancer, miRNA, microRNA, mRNA, messenger RNA, PSA, prostate-specific antigen, mCRPC, metastatic castration-resistant PCa, pri-miRNA, primary miRNA, pre-miRNA, precursor miRNA, dsRNA, double-stranded RNA, EXP5, Exportin-5, PACT, protein kinase R-activating protein, TRBP, trans activating response RNA-binding protein, AGO, Argonaute, RISC, RNA induced silencing complex, CAGRs, cancer-associated genomic regions, LOH, loss of heterozygosity, FRAs, fragile sites, SNPs, single-nucleotide polymorphisms, PTMs, post-translational modifications, TFs, transcription factors, CpG, cytosine-phosphate-guanine, HDAC, histone deacetylase, TGFβR2, transforming growth factor beta receptor 2, PTEN, phosphatase and TENsin homolog, PDCD4, programmed cell death-4, RECK, reversion-inducing cysteine-rich protein with kazal motifs, PIK3IP1, hosphoinositide-3-kinase interacting protein 1, BTG2, B-cell translocation gene 2, NCOR2, AR nuclear receptor corepressor, ARE, androgen response element, RBAK, RB associated KRAB zinc finger, MMP-11, matrix metalloproteinase-11, ADT, androgen deprivation therapy, IL-6, interleukin-6, SLC40A1, Solute Carrier Family 40 Member 1, STK4, serine/threonine kinase 4, Skp2, S-phase kinase-associated protein 2, ROCK2, Coil Containing Protein Kinase 2, PKM2, pyruvate kinase M2, BCL2, B-cell lymphoma 2, CDK6, cyclin-dependent kinase 6, HOXA10, Homeobox A10, MEIS1, Meis Homeobox 1, TGIF2, transforming growth factor beta-induced factor 2 protein, ITGB8, LATS2, Integrin Subunit Beta 8, Large Tumor Suppressor Kinase 2, ZEB2, Zinc Finger E-Box Binding Homeobox 2, PCSCs, prostate cancer stem cells, EZH2, Enhancer of zest homolog 2, Rho, Ras homologous, TRAF1, Tumor necrosis factor receptor associated factor 1, TAB3, TGF-β Activated Kinase 1 (MAP3K7) Binding Protein 3, MAP3K3, Mitogen-Activated Protein Kinase Kinase Kinase 3, FFPE, Formalin-fixed, paraffin-embedded, cDNA, complementary DNA, GSP, gene-specific primer, gDNA, genomic DNA, NGS, Next-generation sequencing, T_m_, melting temperature, BPH, benign prostatic hyperplasia, PBMS, peripheral blood mononuclear cells, AGU, acute genitourinary, AMOs, anti-miRNA oligonucleotides, LNA, Locked nucleic acid, PNA, peptide nucleic acid, CRISPR, Clustered regularly interspaced short palindromic repeats, Cas 9, CRISPR-associated protein 9, PEI, polymer nanoparticle polyethyleneimine, PHB, Poly(3-hydroxybutyrate, PSMA, prostate-specific membrane antigen

## Abstract

Prostate cancer (PCa) is one of the most commonly diagnosed cancers among men worldwide. Despite the presence of accumulated clinical strategies for PCa management, limited prognostic/sensitive biomarkers are available to follow up on disease occurrence and progression. MicroRNAs (miRNAs) are small non-coding RNAs that control gene expression through post-transcriptional regulation of their complementary target messenger RNA (mRNA). MiRNAs modulate fundamental biological processes and play crucial roles in the pathology of various diseases, including PCa. Multiple evidence proved an aberrant miRNA expression profile in PCa, which is actively involved in the carcinogenic process. The robust and pleiotropic impact of miRNAs on PCa suggests them as potential candidates to help more understand the molecular landscape of the disease, which is likely to provide tools for early diagnosis and prognosis as well as additional therapeutic strategies to manage prostate tumors. Here, we emphasize the most consistently reported dysregulated miRNAs and highlight the contribution of their altered downstream targets with PCa hallmarks. Also, we report the potential effectiveness of using miRNAs as diagnostic/prognostic biomarkers in PCa and the high-throughput profiling technologies that are being used in their detection. Another key aspect to be discussed in this review is the promising implication of miRNAs molecules as therapeutic tools and targets for fighting PCa.

## Highlights

• Aberrant expression patterns of miRNAs are reported in prostate cancer and are triggered by multiple genomic anomalies.
• Dysregulated miRNAs are proved to contribute to prostate tumorigenesis by modulating crucial carcinogenic processes.
• High-throughput methods are exploited to profile miRNAs in prostate cancer patients.
• miRNAs’ unique properties make them convenient tools as biomarkers for prostate cancer diagnosis, prognosis, and therapy response assessment.
• miRNAs-based therapeutics are promising tools to reverse the pathological alterations of miRNAs and manage prostate cancer.

## Graphical abstract

**Figure:**
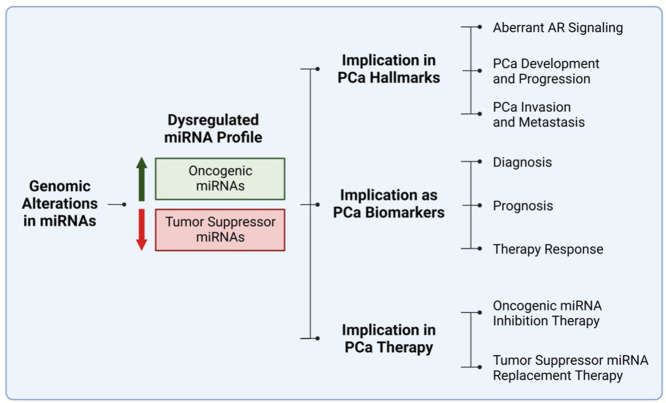

## Introduction

Prostate cancer (PCa) is a multifactorial disease with several factors contributing to its pathophysiology [1]. According to the Global Cancer Statistics (GLOBOCAN) in 2020 produced by the International Agency for Research on Cancer (IARC), PCa accounts for 14.1% of cancer in men, with a mortality rate of 6.8% [2]. In more recent estimates by The American Cancer Society collected by the National Cancer Institute's (NCI's) Surveillance, Epidemiology, and End Results (SEER) program, PCa is the leading type of cancer in men (27% of incidence) and is responsible for 11% of cancer-related deaths in the United States [3]. This rise in incidence and mortality rate is linked with the depression of healthcare systems due to Corona Virus 2019 (CoVid-19), impeding diagnosis and treatment temporarily [4].

The classification of PCa is widely determined using the American Joint Committee TNM system, which takes into consideration both clinical and pathological staging [5]. Clinical staging assesses the primary tumor using a digital rectal examination, a prostate-specific antigen (PSA) test, and imaging modalities. Meanwhile, pathological staging takes into consideration the histological examination of a transrectal ultrasound (TRUS)-guided biopsy [6]. Determining both, the stage and grade, facilitate the therapeutic journey permitting the physician and patient to weigh out the risk and benefits of each therapeutic regimen. Traditionally, in localized PCa, with low to intermediate risk, watchful waiting, active surveillance, surgery (radical prostatectomy), and radiation therapy are opted for. However, for locally advanced and metastatic disease stages, androgen suppression and chemotherapeutic agents are considered [7]. Emerging novel therapeutic modes hold promise when it comes to both primary and metastatic PCa, owing to a better understanding of PCa oncogenesis, and advancements in the diagnosis and treatment personalization [8]. These include diagnosis, prognosis, predictive, and treatment monitoring response by human biofluid circulating microRNA (miRNA) [9], diagnosis using molecular imaging (PET-CT) [10], targeted radioisotopes for metastatic castration-resistant PCa (mCRPC) [11], and immunotherapy [12]. miRNAs are a class of single-stranded small non-coding RNA (∼19-24 nucleotides in length) that negatively regulate complementary messenger RNA (mRNA), ultimately affecting its protein expression [13]. Globally, miRNAs are a topic of interest as they play crucial roles in cell physiology and cancer pathophysiology, including PCa. There are several miRNA molecules implicated in the regulation of apoptosis, tumor growth, metastasis, and drug resistance. Thus, the initiation and progression of neoplastic diseases can be modulated by these small molecules [14,15]. This renders them targets for diagnostic, prognostic, and therapeutic agents, and developments in this field are surging [9,[16], [17], [18], [19]].

Here we overview the implication of miRNAs in PCa tumorigenesis and the significance of their usage as biomarkers. Also, we discuss the high-throughput screening technologies and the promising therapeutic approaches that can be potentially used as tools against PCa.

## miRNAs biogenesis

**Fig. 1:**
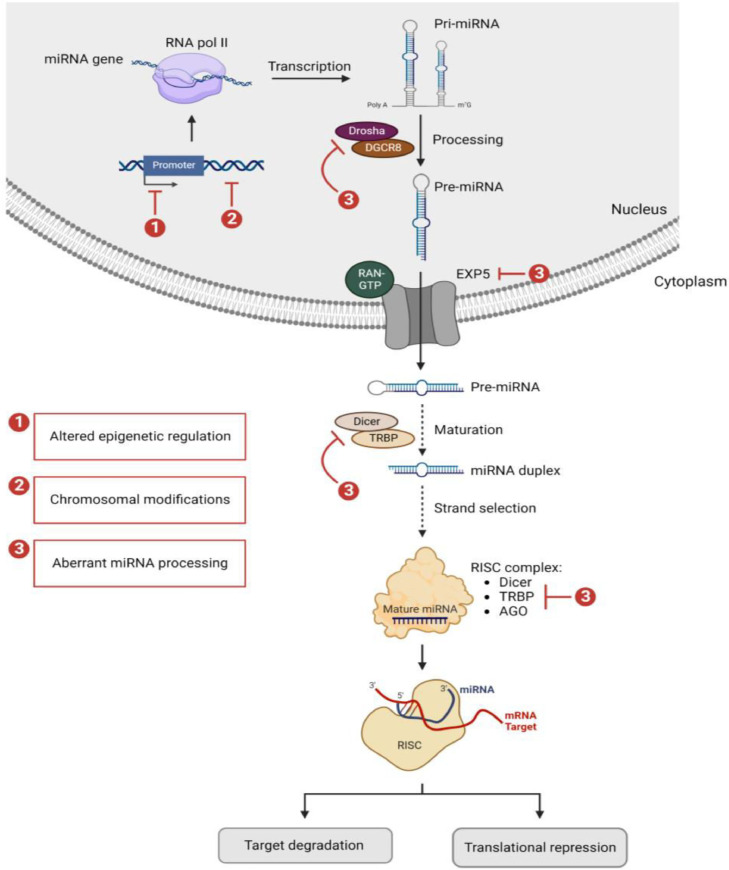
Schematic representation of miRNA biogenesis. miRNA genes are transcribed by RNA pol II to generate long pri-miRNAs. The latter are cleaved by a Microprocessor complex, which includes DROSHA and DGCR8, and produce a 60–70-nucleotide pre-miRNAs. The pre-miRNAs are exported from the nucleus to the cytoplasm by XPO5 and further processed by DICER1 to produce mature miRNA duplexes. The mature guide strand is selected and loaded into RISC, which contains DICER1, TRBP, and AGO proteins. The guide strand directs RISC to the mRNA targets resulting in either mRNAs degradation or translational repression. Alterations at the level of miRNA gene itself or its promoter and the biogenesis core processing enzymes can occur resulting in a dysregulated miRNA expression profile. Abbreviations: miRNA; microRNA, RNA pol II; RNA polymerase II, pri-miRNAs; primary miRNAs, DGCR8; DiGeorge syndrome critical region 8, pre-miRNAs; precursor miRNAs, XPO5; exportin 5, RISC; miRNA-induced silencing complex, TRBP; transactivation-responsive RNA-binding protein, AGO; Argonaute, mRNA; messenger RNA.

MiRNA molecules serve as guiding molecules for regulating gene expression. A single miRNA can target and regulate hundreds of mRNAs, which implicate them in almost all physiological and cellular processes [20]. The biogenesis of miRNAs is under tight temporal and spatial control. It is a rigorous processing pathway that ensures only RNAs with the correct structures and sequences can regulate gene expression [21,22]. The multistep miRNA biogenesis cascade includes miRNA gene transcription, primary miRNA (pri-miRNA) processing, pri-miRNA export to the cytoplasm, precursor miRNA (pre-miRNA) maturation, and transcript targeting (refer to Fig. 1).

The first step in miRNA biogenesis involves the transcription of miRNA gene by RNA polymerase II, which generates a 5′ capped and 3′ polyadenylated pri-miRNA transcript [20,23]. The pri-miRNA is then cleaved inside the nucleus by a Microprocessor complex, releasing a small hairpin structure known as pre-miRNA [24,25]. The Microprocessor complex comprises the RNase III enzyme DROSHA and its essential cofactor, the double-stranded RNA (dsRNA)-binding protein DiGeorge syndrome critical region 8 (DGCR8) [26].

Next, the pre-miRNA is exported to the cytoplasm by Exportin-5 (EXP5), a RAN-GTP dependent nucleo/cytoplasmic cargo transporter [27]. In the cytoplasm, the type-III RNAse DICER further cleaves the pre-miRNA into a short dsRNA duplex of around 22 nucleotides. This duplex contains a mature guide miRNA strand and its complementary passenger strand [28]. To effectively cleave the pre-miRNA, DICER forms a complex with other proteins such as the protein kinase R-activating protein (PACT) and the Trans activating Response RNA-Binding Protein (TRBP) [29]. Afterward, the guide and passenger strands of the miRNA duplex are loaded into the Argonaute (AGO) proteins to create the RNA Induced Silencing Complex (RISC). Dicer, TRBP, PACT, and AGO proteins are all components of RISC [30]. The mature guide strand, with the lower thermodynamic stability at its 5′-end, is then selected and the corresponding passenger strand is discarded as part of an ATP-independent miRNA duplex unwinding process inside the AGO proteins [31,32]. After which, the miRNA directs this catalytic complex to a particular mRNA sequence to negatively regulate its expression. Depending on the base-pairing complementarity between the guide miRNA and its mRNA target, the latter is either cleaved and degraded or translationally inhibited [29].

## miRNAs dysregulation in cancer

Given the importance of miRNAs in regulating gene expression and modulating a plethora of cellular processes, it is not surprising that their dysregulation is associated with various pathological diseases. Aberrant expression patterns of miRNAs are reported in a wide spectrum of human cancers including PCa [33,34]. This dysregulation is triggered by several genomic anomalies including, chromosomal modifications, aberrant miRNA biogenesis, and altered epigenetic regulation (refer to Fig. 1).

### Chromosomal modifications

Alterations in miRNA-containing genomic loci and their associated genes have been proved to be partly responsible for miRNA dysregulation in cancer [35]. In a genome-wide systematic study for correlations between the genomic positions of miRNA genes and cancer, Calin et al. [36] showed that cancer-related miRNA genes are non-randomly distributed among the human chromosomes but concentrated in certain chromosomes. MiRNA genes preferentially reside in particular genomic regions that are prone to alteration in cancer cells, known as cancer-associated genomic regions (CAGRs). CAGRs can take the form of (i) minimal common regions of loss of heterozygosity (LOH), which are thought to harbor tumor-suppressor genes, (ii) minimal common regions of amplification (minimal amplicons), which are thought to have oncogenes, (iii) common breakpoint regions, and (iv) fragile sites (FRAs) for sister-chromatid exchange, translocation, or integration of plasmid DNA and tumor-associated viruses [37,38]. Moreover, single-nucleotide polymorphisms (SNPs) in miRNA genes also alter their expression levels and affect their functional role in mediating gene regulation, thereby affecting cancer risk [39], [40], [41], [42].

### Aberrant miRNA biogenesis

As elaborated in the previous section, different components participate in miRNA biogenesis cascade and ensure the correct maturation of pri-miRNA. The level of expression and activity of the core processing enzymes, along with other regulatory factors, are often found dysregulated in cancers. This dysregulation is accompanied by aberrant miRNA biogenesis and accordingly an altered miRNA expression profile [43,44]. As evidenced in the literature, described mutations in the microprocessor components, DROSHA and its co-factor DGCR8, trigger impairments in the pri-miRNAs nuclear processing [45], [46], [47]. Abnormal expression and/or activity of EXP5 protein interrupt the pri-miRNAs export to the cytoplasm [48]. Defects in the DICER/TRBP processing complex disrupt miRNAs maturation processing [47]. Changes in the stability and/or activity of AGO proteins, mainly AGO2, alter the post-translational modifications (PTMs) of miRNAs and critically affect miRNA gene-silencing function [49]. As well, aberrant expression of some key regulatory factors, such as RNA binding factors and transcription factors (TFs; activators or repressors), significantly prompts faults in miRNA biogenesis and processing cascade [50], [51], [52], [53]. Accordingly, such defects alter the global miRNA expression level and increase the susceptibility to oncogenic shifts.

### Altered epigenetic regulations

Like other coding and non-coding genes, miRNAs are subjected to epigenetic regulations which control the DNA methylation and histone modification patterns at their genomic loci [54]. Impaired epigenetic regulations induce functional changes resembling that of genetic mutations and result in aberrant miRNA expression in cancer [55,56]. Indeed, epigenetically regulated miRNAs are proved to be dysregulated in the pathogenesis of various cancers [57], [58], [59]. In an integrated review of 150 papers, 6.9 % of the total known mature miRNAs were shown to be regulated by DNA methylation in 36 different cancer types [60]. This is explained by the fact that one-third of human miRNAs have cytosine-phosphate-guanine (CpG) dinucleotides rich regions, or CpG islands, in their upstream promoter. These islands represent hotspots for methylation and tightly correlate miRNAs gene expression with the DNA methylation status [61,62]. The epigenetic silencing of tumor-suppressor miRNAs by promoter-associated CpG hyper-methylation is a common hallmark of human tumors [63]. For instance, the epigenetic disruption of the tumor suppressor miR-130a in PCa, mediated by the hypermethylation of its promoter region, is proved to promote key molecular and phenotypic features of prostate carcinogenesis [64]. A similar miRNA-mediated epigenetic cross talk occurs with key histone modifications. The latter modulate the chromatin structure and accessibility at gene loci and hence regulate both the activation and repression of miRNA expression [65,66]. Altered histone modifications, such as those mediated by histone deacetylase (HDAC) and polycomb repressor complexes (PRC1 or PRC2) overexpression, have been identified in cancer in association with dysregulated miRNA profile. A combination of chromatin immune-precipitation (ChIP)-on-chip and miRNA microarray analysis in PCa cells revealed that miRNA expression correlates positively with histone 3 lysine 4 tri-methylation (H3K4me3) and correlates inversely with histone 3 lysine 27 tri-methylation (H3K27me3) in miRNA promoter regions [67]. For example, the overexpression of Enhancer Of Zeste 2 Polycomb Repressive Complex 2 Subunit (EZH2), which tri-methylates H3K27, leads to the silencing of multiple tumor suppressive miRNAs in PCa [68].

## MiRNAs implication in PCa hallmarks

**Fig. 2:**
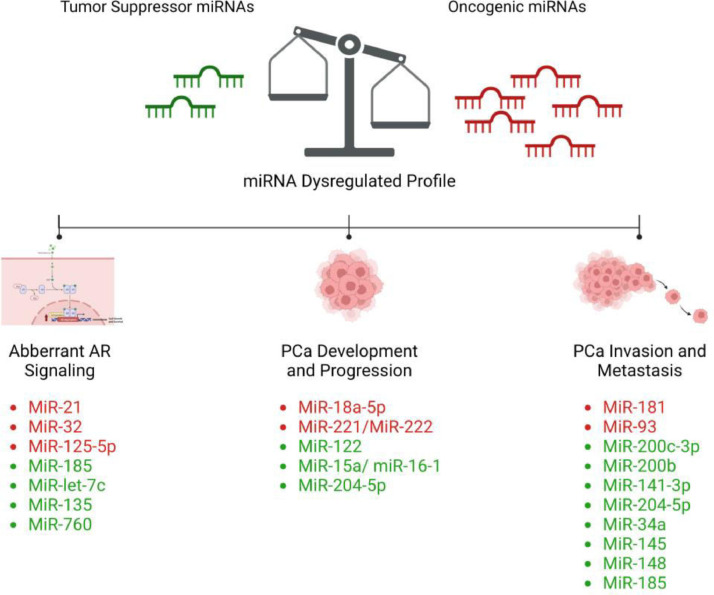
Schematic representation of the most frequent dysregulated miRNAs in PCa. Dysregulated miRNA profile is associated with aberrant AR signaling, tumor development, progression, invasion, and metastasis in PCa. MiRNAs in red represent the up-regulated oncogenic miRNAs and those in green represent the down-regulated tumor suppressor miRNAs. Abbreviations: miRNA; microRNA, AR; androgen receptor, PCa: prostate cancer, MiR; microRNA.

Dysregulated miRNAs have been shown to contribute to prostate tumorigenesis via the loss of tumor-suppressing miRNAs or amplification of tumor-promoting/oncogenic miRNAs [69]. Numerous studies have validated the association between the dysregulated miRNAs and the initiation of PCa toward the acquisition of metastatic phenotype, by modulating crucial processes such as androgen receptor (AR) signaling, proliferation, apoptosis, epithelial to mesenchymal transition (EMT), and metastasis [[70], [71], [72]] (refer to Fig. 2).

### MiRNAs association with AR signaling

It is well-established that alterations in AR signaling critically derives the growth and progression of both localized and advanced metastatic PCa. Several miRNAs are proved to interact with AR mRNA and regulate its expression, either by binding directly to the mRNA of AR itself or AR-associated factors, or indirectly by affecting the expression of AR co-regulators [73].

MiR-21 is one of the most widely studied miRNAs in cancer [74,75]. It is overexpressed in a variety of solid tumors, including PCa, and exhibits predominantly an oncogenic role in disease occurrence and development [76,77]. MiR-21 itself is a direct transcriptional target of AR, which in turn, increases AR expression in PCa, thus suggesting the existence of a regulatory loop [78]. MiR-21 exerts its effect potentially by targeting and inhibiting several tumor suppressor genes, including transforming growth factor beta receptor 2 (TGFβR2) [79], phosphatase and TENsin homolog (PTEN) [80], the translational inhibitor programmed cell death-4 (PDCD4) [81], and the membrane-anchored protease-regulator reversion-inducing cysteine-rich protein with kazal motifs (RECK) [82], thus supporting PCa promotion and progression. In addition, several studies evidenced miR-32 to be consistently dysregulated miRNAs in PCa, with an overexpression being detected especially at the CRPC stage [83,84]. MiR-32 is an AR-regulated miRNA and it contributes to PCa cell growth and progression by blocking the expression of the tumor suppressor genes phosphoinositide-3-kinase interacting protein 1 (PIK3IP1) and B-cell translocation gene 2 (BTG2), and favoring the PI3K/AKT/mTOR pathway [84]. Moreover, miR-125b-5p is a well-described oncogenic miRNA in PCa involved in AR signaling. MiR-125b-5p acts indirectly via modulating the AR nuclear receptor corepressor (NCOR2) [85].

Besides, a study on PCa cells identified binding sites for miR-185 in the 3′UTR of AR mRNA. Remarkably, the blockade of AR transcripts by miR-185 showed to compromise the interaction between AR and androgen response element (ARE) and decrease the level of AR target genes' expression, hence suggesting miR-185 as a negative regulator of AR signaling [86]. MiR-let-7c is another tumor suppressor miRNA that antagonizes AR expression and activity indirectly by targeting c-Myc [87]. MiR-let-7c is found to be down-regulated in PCa specimens and to be associated with disease progression [88,89]. Moreover, Wan et al. showed that the androgen-induced miR-135a acts as a tumor suppressor in PCa mainly through modulating the expression of RB associated KRAB zinc finger (RBAK) and matrix metalloproteinase-11 (MMP-11). MiR-135a down-regulation causes loss in tumor-suppressive activities and mediates PCa progression in response to androgen deprivation therapy (ADT) [90]. Furthermore, miR-760 was proven recently to be expressed at low levels in PCa tissues compared to normal tissues. As an androgen-regulated tumor suppressor miRNA, the attenuation of miR-760 induces the proliferation and growth of PCa cell lines, primarily by targeting and inhibiting the expression of interleukin-6 (IL-6) [91].

### MiRNAs association with PCa development and progression

Among promising miRNAs explored, miR-18a which belongs to the miR-17–92 cluster is upregulated in PCa and functions as a tumor promoter [92,93]. MiR-18a-5p overexpression stimulates the proliferation of PCa cells by targeting the iron transporter Solute Carrier Family 40 Member 1 (SLC40A1) [94]. Also, miR-18a-5p showed to attenuate the expression of the pro-apoptotic protein serine/threonine kinase 4 (STK4), consequently resulting in an increased AKT phosphorylation and enhanced tumor cell survival [95]. As well, the miR-221/miR-222 oncogenic cluster is found at high levels in PCa. One proposed mechanism for miR-221/miR-222 involves attenuating the expression of p27kip1, and in turn affecting the expression of several genes involved in cell cycle and proliferation, including cyclin D1, cyclin A, and S-phase kinase-associated protein 2 (Skp2) [96,97].

Moreover, miR-122 serves as a tumor suppressor miRNA in PCa pathogenesis. Its downregulation is linked with increased proliferation and upregulation of Rho Associated Coiled-Coil Containing Protein Kinase 2 (ROCK2) [98]. In another study, decreased levels of miR-122 showed to be accompanied by increased proliferation, inhibited apoptosis, and PCa resistance to docetaxel, potentially through targeting the expression of enzyme pyruvate kinase M2 (PKM2) [99]. In addition, the loss of miR-15-a-miR-16-1 tumor suppressor cluster showed to affect PCa proliferation and survival by targeting multiple genes such as B-cell lymphoma 2 (BCL2), cyclin D1, cyclin E1, and cyclin-dependent kinase 6 (CDK6) [100]. miR-204-5p is an additional well-documented tumor suppressor, which affects PCa growth by controlling the expression of the Homeobox A10 (HOXA10) and Meis Homeobox 1 (MEIS1), as well as the anti-apoptotic gene BCL2 [101,102].

### MiRNAs association with PCa invasiveness

Zhiping et al. reported the expression of miR-181a to be at higher levels in metastatic prostate tumor samples compared with primary prostate tumors. The overexpression of miR-181a showed to contribute to EMT phenotype by downregulating the expression of the epithelial marker E-cadherin and upregulating that of the mesenchymal markers N-cadherin, vimentin, and snail. MiR-181 overexpression was also shown to promote PCa cell migration and invasion by directly targeting transforming growth factor beta-induced factor 2 protein (TGIF2) [103]. As well, it contributes to the resistance of PCa cells to docetaxel and cabazitaxel, in part by modulating p53 phosphorylation and apoptosis [104]. As a member of the miR-106b-25 cluster, the expression level of miR-93 is also found up-regulated in PCa. Its role in disease progression involves increasing the expression levels of TGFβR2, Integrin Subunit Beta 8 (ITGB8), and Large Tumor Suppressor Kinase 2 (LATS2). Moreover, miR-93 significantly correlates with Gleason score, lymph node involvement, bone metastasis, and TNM stage in PCa [105,106].

Besides, the tumor suppressor miRNAs of miR-200 family are critical regulators of EMT. This family includes miR-200a/b/c, miR-429, and miR-141. In a study done to investigate the role of miR-200c-3p in PCa invasiveness, miR-200c-3p was found to be significantly down-regulated in human PCa cell lines (PC3 and DU145) compared with the normal prostatic epithelial cell line (RWPE1). MiR-200c-3p is proposed to inhibit cell migration and invasion via targeting the E-cadherin repressor and EMT inducer; Zinc Finger E-Box Binding Homeobox 2 (ZEB2) [107]. In line, the downregulation of miR-200b in PCa showed to inhibit EMT, growth, and metastasis via a similar ZEB1-mediated pathway [108]. Similarly, miR-141-3p downregulation showed to participate in PCa metastasis and invasiveness via activating NF-κB signaling [109]., it showed to enrich the stemness characteristics of prostate cancer stem cells (PCSCs) by targeting a cohort of pro-metastasis genes including CD44, Enhancer of zest homolog 2 (EZH2), and Ras homologous (Rho) GTPases [110]. In addition to its role in modulating growth and apoptosis, miR-204-5p downregulation is also associated with disease progression and metastasis. Wa et el. showed miR-204-5p to repress invasion, migration, and bone metastasis by inhibiting nuclear factor κB (NF-κB) signaling via simultaneously targeting Tumor necrosis factor receptor associated factor 1 (TRAF1), TGF-β Activated Kinase 1 (MAP3K7) Binding Protein 3 (TAB3), and Mitogen-Activated Protein Kinase Kinase Kinase 3 (MAP3K3) [111]. Moreover, multiple studies elucidated the critical role of miR-34a tumor suppressor in PCa invasiveness, and downregulated level of miR-34a has been observed in PCa tumors [112]. Liang et al. [113] found miR-34a to negatively regulate the Wnt signaling pathway and inhibit EMT-associated migration and invasion in PCa. Also, liu et al. [114] showed that miR-34a mediates the paclitaxel-based chemotherapy resistance in PCa cells via direct suppression of the JAG1/Notch1 axis. As well, Yan et al. [115] demonstrated the role of miR-34a in modulating PCSCs and metastasis by directly repressing CD44 expression. Furthermore, a recent review discussed extensively the involvement of other miRNAs, including miR-145, miR-148, and miR-185, in regulating the behavior of PCSCs and contributing to PCa invasiveness and metastasis [116].

## MiRNAs as potential biomarkers in PCa

### MiRNAs high-throughput detection methods

As defined by the National Cancer Institute (NCI), a biomarker is a molecule detected in the body fluids or tissues indicating a normal or abnormal process, condition, disease, or even treatment response. A number of established biomarkers exist in the literature of which miRNAs emerged as potential candidates for cancer diagnosis, prognosis, and therapy selection and assessment [[117], [118], [119]]. MiRNAs are advantageous due to their considerable stability, and high resistance to ribonucleases as well as harsh physiochemical conditions that normally lead to RNA degradation [[119], [120], [121], [122]]. Moreover, expression levels of miRNAs display an organ and tissue-specific profile. They are dysregulated within specific cancer types and differentially expressed between a tumor and its corresponding normal tissue [123,124]. In addition, miRNAs may be extracted from various sources. For PCa, besides conventional tissue biopsies (formalin-fixed, paraffin-embedded (FFPE), and fresh-frozen), samples may be collected less invasively and repeatedly from blood (serum, plasma, exosomes), urine, and semen [[125], [126], [127]].

These facts, among others, made miRNAs attractive molecules to explore as biomarkers and employ in routine clinical practice. To fulfill this purpose, miRNAs are being profiled using suitable and powerful technologies that were developed and enhanced over the years to overcome the challenges inherited by the nature of these molecules [128]. Here, we describe the current approaches for high-throughput detection of miRNAs in PCa with emphasis on their advantages and their limitations (refer to Fig. 2 and Table 1).

**Table 1:** Main high-throughput approaches for miRNAs profiling, comparing their features and limitations in detecting these molecules.

Detection methods	Principle/Main functions	Advantages	Limitations
qRT-PCR	- Amplification-based - Best used for validation of targets obtained by large-scale approaches	- Convenient/simple - Relatively fast - Relatively inexpensive - Relatively easy data analysis - High sensitivity and specificity - Absolute and relative quantification	- Challenges in primers design - Controversial reference genes - Difficulty to discern subfamily targets - Sensitive to gDNA contaminants
Microarrays	- Hybridization-based - Best used for preliminary screening	- Relatively fast - Easily standardized - Biochips readily available	- Low sensitivity - Low specificity for targets in the same subfamily - Cross-hybridization and background noise issues - Restricted to array content - Relatively expensive custom-made chips
Deep sequencing	- Sequencing-based - Best used for large-scale novel biomarkers identification	- Detection of novel targets - High dynamic range, sensitivity, and, specificity - No prior sequence knowledge required - Absolute quantification	- Relatively high cost - Complex data processing and interpretation - Requirement of specialized personnel and equipment - Sequencing bias - Time-consuming
Nanostring's nCounter	- Hybridization-based	- No amplification steps - Relatively fast - Absolute quantification - High accuracy	- Only available in specialized facilities with limited accessibility - Reduced sensitivity

Abbreviations: qRT-PCR; quantitative real-time reverse-transcription-polymerase chain reaction, gDNA; genomic DNA.

#### Quantitative real-time reverse-transcription-polymerase chain reaction (qRT-PCR)

**Fig. 3:**
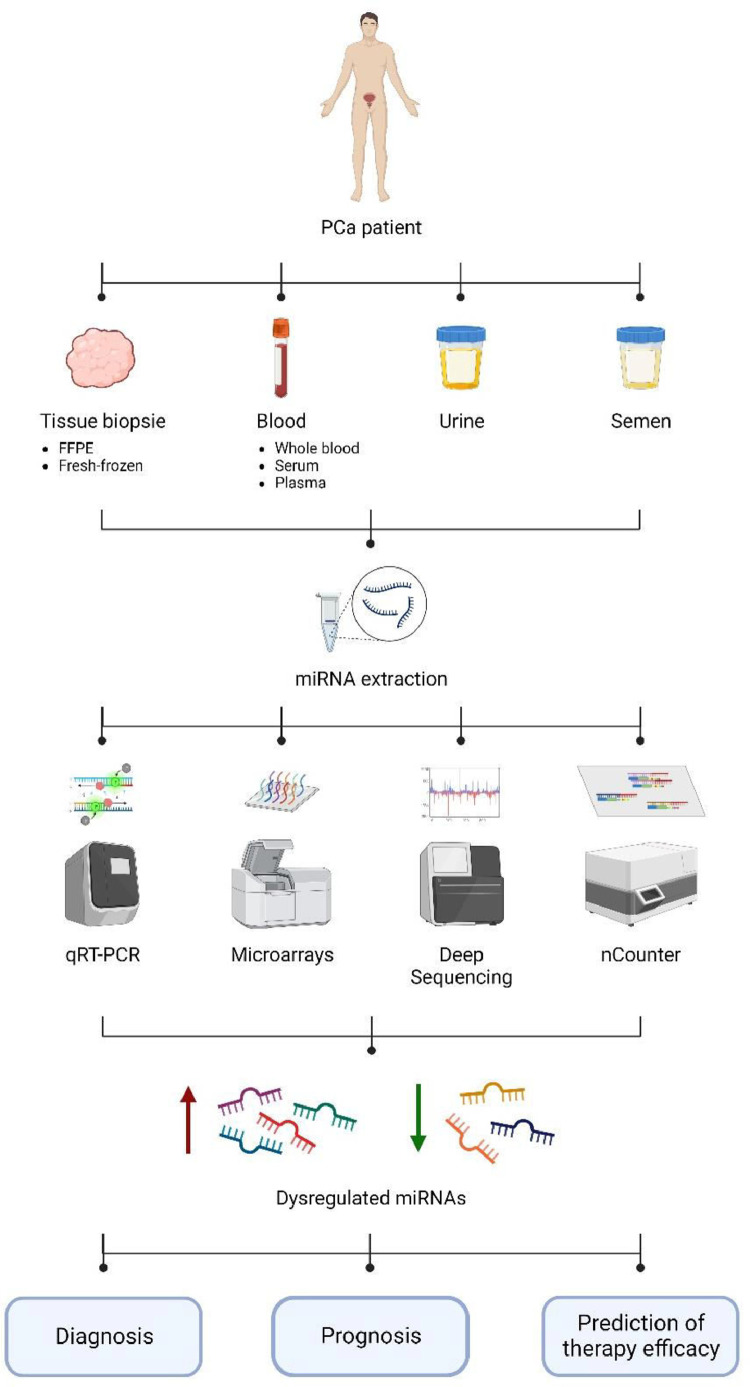
Schematic representation of the different high-throughput tools for miRNAs detection in PCa. MiRNAs may be extracted from various sources such as tissue biopsies, blood, urine, and semen. Several high-throughput strategies exist to detect miRNAs. They range from qRT-PCR, microarray, and next-generation sequencing to NanoString nCounter-based methods. These tools are useful to identify the miRNAs with a dysregulated profile which may consequently serve as biomarkers for PCa diagnosis, prognosis, and response to therapy. Abbreviations PCa; prostate cancer, FFPE; formalin-fixed paraffin-embedded, miRNA; microRNA, qRT-PCR; quantitative real-time reverse-transcription-polymerase chain reaction.

qRT-PCR is considered a gold standard technique for the quantification of miRNAs expression [[129], [130], [131], [132]]. In fact, this method has been widely used in PCa to detect miRNAs from tissues and/or bodily fluids [[130], [131], [132]]. It is a temperature-dependent target amplification-based method where the miRNA is first reverse transcribed into its complementary DNA (cDNA), which is subsequently exponentially amplified and quantified by PCR allowing real-time fluorescence detection. Since mature miRNA are not polyadenylated, cDNA synthesis is performed mostly using the stem-loop primer, poly(T) adapter, or gene-specific primer (GSP). Following RT step, miRNA quantification may be commonly achieved using either SYBR Green fluorescent dye or TaqMan probe. SYBR Green dye is cost-effective, while TaqMan probe is more specific [128,[133], [134], [135], [136], [137]]. qRT-PCR is a convenient, simple, rapid, and inexpensive technique with a relatively easy data analysis. It is highly specific and sensitive capable of detecting a very low target copy number, which is important given the low abundance of some miRNAs in the clinical samples [[138], [139], [140]]. In addition, qRT-PCR may be scaled up and thus, used as a high-throughput method allowing the screening of up to 384 miRNAs in one run [[141], [142], [143]]. It can also be employed to validate results from other genome-wide profiling techniques [[144], [145], [146]]. However, this methodology presents some limitations when it comes to miRNAs quantification, noting some in the following section. MiRNAs in bodily fluids are in general scarce compared to the genomic DNA (gDNA), thus, eliminating all gDNA contaminants from the clinical samples prior RT is very crucial. Designing suitable primers for miRNAs detection is challenging since the pre-miRNAs are in the form of a stable hairpin, while the mature miRNAs are of short length almost the size of the PCR primers. Moreover, miRNAs belonging to the same family may differ by only one nucleotide making it hard to discern them. Also, Choosing the right endogenous reference genes, allowing data normalization, remains contentious with no widely accepted inner control genes [128,[147], [148], [149]]. Since qRT-PCR is a multi-step technique, it is more prone to error and false positive results. Its output depends highly on the quality of the input RNA from the clinical samples. Also, laborious experimental conditions related to the nature of miRNAs or to the technique itself such as target elongation, cDNA synthesis, and amplification steps all form potential sources of bias [150,151]. In general, qRT-PCR is best used to validate screening results from other genome-wide approaches (Fig. 3).

#### Microarrays

Microarray-based technique is widely used for the profiling of identified miRNAs and is commonly employed in PCa studies [88,[152], [153], [154]]. It is a high-throughput method that allows large-scale detection of miRNAs along the changes in their expression levels in one experiment. Microarray is a multistep hybridization-based technique whereby target molecules bind to their complementary probes that are fixed on impermeable solid supports. Extracted miRNAs in a particular sample are labeled with one of the various fluorescent dyes that may be used such as Alexa dyes, Cy3, and Cy5. As previously mentioned, miRNAs lack a poly(A) tail, thus, in contrast with the mRNAs, they cannot be labeled using the poly(T) RT protocol. Rather, miRNAs may be conjugated directly with the fluorescent dye or indirectly whereby the miRNA transcripts or their corresponding RT-PCR products may be labeled instead of the miRNAs. The labeling step allows the miRNAs that bind to their corresponding probes to emit fluorescence at precise recorded locations on the solid chip. Accordingly, the detected fluorescence signal intensity reflects the presence of particular miRNAs and their relative expression levels in the tested sample. This method may also be used to compare the miRNAs profile between two samples using two different fluorescent dyes with different emission wavelengths (two-color microarray) [[155], [156], [157]]. Microarray-based method is particularly attractive for several factors. It is rapid and easily standardized. Most importantly, it allows high-throughput genome-wide screening of miRNAs from different samples simultaneously. Consequently, it offers the benefit of comparing miRNAs profiles between different conditions, organs, and tissues. In fact, using microarray-based method, miRNA profiles from hundreds of tumors and normal tissues were established, presenting a global miRNAs signature for several types of cancer [156,158]. Besides custom-made microarrays, some chips are readily available as miRNA commercial platforms are designed by many companies. Thus, biochips are attractive tools to be employed for biomarkers detection for clinical applications [128,159]. On the other hand, microarray-based method presents some drawbacks. MiRNAs with a very short length, low abundance, and similar sequences may not be detected since this technique is limited by the low sensitivity, low specificity for miRNAs in the same subfamily, cross-hybridization, and background noise problems. This technique requires prior knowledge of the miRNA sequences and is restricted by the array content; thus, discovery of novel miRNAs using this method is not feasible. In general, microarray-based technique is best used for preliminary screening of miRNAs [133,147,149,160].

#### Deep sequencing

Next-generation sequencing (NGS)-based methods are powerful tools to screen whole-genome miRNAs and are being extensively used in various systems including PCa [[161], [162], [163]]. These are high-throughput methods that can produce millions of reads in parallel allowing the profiling of known miRNAs as well as the discovery of new ones. In fact, NGS-driven techniques led to the identification of most of the miRNAs [128,164]. These methods start with a wet experiment part whereby RNAs are extracted, then miRNAs are isolated from the total RNAs pool. Subsequently, sequencing adaptors are ligated at the 5’ and 3’ ends of the miRNAs. The latter are then subject to RT and PCR amplification. These steps are followed by the sequencing part that depends on the platform used (Illumina Genome Analyzer, SOLiD™, and Ion Torrent). Consequently, millions of short reads are obtained. The dry experiment part i.e. data processing starts with the alignment of all resulting reads to the latest corresponding genome (human genome) or miRNA sequences represented on miRBase (the microRNA database; https://www.mirbase.org/). Accordingly, bioinformatics analysis allows the identification and quantification of established and novel miRNAs and their variants such as isomers [[164], [165], [166], [167]]. The main feature of NGS is its very high-throughput sequencing and sample multiplexing where large numbers of targets are pooled and sequenced in parallel using individual “barcodes” (indexes) that flag the sources of the samples [167]. Deep sequencing-based methods present several advantages compared to other high-throughput techniques such as microarrays. As previously mentioned, these methods allow the detection of not only known miRNAs but also unexplored ones. Problems with background noise and cross-hybridization are less pronounced in NGS techniques. Moreover, the latter has a high dynamic range allowing precise quantification of the abundant miRNAs as well as the ones with low expression levels simultaneously in one run. In fact, NGS-based methods are highly-accurate due to the high depth of coverage of each base by the aligned short reads. They are highly sensitive and specific; capable of detecting differences in 1 bp of miRNA allowing the discrimination between miRNAs in the same subfamily. Finally, NGS-based methods can give an absolute quantification of the miRNAs [165,[168], [169], [170]]. Nevertheless, these techniques are still limited by several factors. The cost of performing deep sequencing is still relatively high impeding its use in routine lab work. Data processing and interpretation are complex requiring time along with specialized personnel and equipment. For these reasons, sequencing is not yet suitable for rapid clinical tests. Despite remarkable improvements, sequencing bias, which may be introduced at different experimental steps, still exists such as issues inherited by the PCR amplification and adapters’ ligation [150,170,171]. In general, NGS-based techniques are best used for large-scale novel miRNAs discovery and new biomarkers identification [150].

#### NanoString nCounter

nCounter Analysis Systems by Nanostring are newly developed, digital-quantification, amplification-free methods that are being increasingly used in PCa [[172], [173], [174]]. These platforms allow the direct quantification of native miRNAs from their sources with no need for RT, amplification, or technical replicates. They are suitable for multiplex analysis of more than 800 targets. Since miRNAs are short with variable sequences, proper multiplexing is ensured by adding and ligating miRTags to the miRNAs. This unifies the melting temperature (T_m_) of all targets where subsequently the hybridization is performed at the same temperature regardless of miRNAs sequence. Following these steps, capture and reporter probes are hybridized to the corresponding miRNAs of interest forming unique target-probe complexes or codesets. The capture probes immobilize the codesets on the imaging surface in the nCounter Cartridge, whereas the reporter probes allow the identification of the miRNAs of interest through specific barcodes (color codes) of 6 molecules formed by the combination of 4 fluorophores. Subsequently, the sample is scanned by a digital analyzer and the targets are directly counted and identified by their unique color codes [147,169]. nCounter platforms have several features. This technique is devoid of amplification or cloning steps which may reduce the possibility of bias. Moreover, it is a relatively fast technique. It enables the measurement of a wide range of targets in parallel and provides absolute quantification of the miRNA levels. Also, it has a high accuracy being able to differentiate between similar miRNAs. However, nCounter platforms are not yet suitable for routine clinical practices and are only available in specialized facilities with limited accessibility. They also showed reduced sensitivity compared to other platforms such as NGS [169,175,176].

Third-generation sequencing platforms such as nanopore single-molecule technology and its variants are novel, rapidly evolving strategies that are reported in few studies working on PCa. Based on recent work that validated the efficiency of nanopore-based techniques to detect particular miRNAs, these approaches seem promising in screening these molecules noninvasively while avoiding any sample processing and amplification steps. Interestingly, these can be engineered allowing the detection of multiple miRNAs targets at the same time. Consequently, future studies may validate the feasibility of implementing these approaches for high-throughput screening, particularly in clinical practices [[177], [178], [179], [180]].

In a conclusion, no definitive technique for miRNA detection that is suitable for routine clinical practices exists yet. All current, as well as new emerging approaches, have advantages and limitations that need to be considered for the study design. Obtained results require meticulous analysis and assessment since conclusions may differ depending on the platform used.

### miRNAs as biomarkers for diagnosis, prognosis, and therapy response

MiRNA translation into clinical practice can be a convenient tool in the determination of PCa diagnosis, prognosis, and therapeutic response. PSA remains the primary diagnostic test for PCa, however, its specificity is limited which often leads to over-diagnosis [181]. Its diagnostic accuracy can be improved by miRNA analysis as their dysregulation has been shown to be indicative of pathology and can differentiate between non-malignant pathophysiology, such as benign prostatic hyperplasia, and malignant disease [182].

Numerous miRNAs have been correlated with PCa diagnosis, however, only a few have been identified in more than one study. In this regard, Porzycki et al. reported that miR-106b, miR-141-3p, miR-21, and miR-357 were significantly overexpressed in the serum levels of PCa patients when compared to healthy control patients with 93% sensitivity and 63% specificity in the prediction of PCa [183]. In another study by Haldrup et al., the overexpression of serum miR-141-3p and miR-357 was noted in PCa patients compared to patients with benign prostatic hyperplasia (BPH) [184]. In a study analyzing miR-21, miR-141, and miR-221 levels in the blood circulation of healthy individuals, patients diagnosed with local PCa, and patients with bone metastatic PCa, an increase in the levels of miR-21 and -221, and no variation in miR-141 levels was noted. However, an increase in miR-141 levels was the most significant indication of metastatic PCa [185]. Therefore, miR-21 has been identified as a more convenient PCa biomarker while miR-141 has been more applicable in discerning localized or locally advanced PCa from metastatic PCa as a prognostic tool, supported by other studies [[186], [187], [188]]. Early detection of PCa was shown to be potentially facilitated through the use of a plasma miRNA panel demonstrated by Matin et al. The panel comprising four miRNAs (miR-98-5p, miR152-3p, miR-326, and miR-4289) was shown to be overexpressed in PCa patients compared to healthy controls. This panel was shown to improve the early detection of PCa [189]. Liu et al. developed a score depending on serum levels of a panel consisting of 3 miRNAs, miR-223, miR-24, and miR-375, to compare PCa patients that require active surveillance from patients that require treatment due to disease progression [190]. The analysis of PCa and non-cancerous tissues revealed the deregulation of >100 miRNA in PCa as compared with non-cancerous prostate tissue. After the selection of 4 miRNAs to be analyzed in urine samples, in both cohorts, miR-148a and miR-375 were identified as specific biomarkers of PCa enhancing the diagnostic value of the PSA test [191].

As mentioned previously, miRNA dysregulation can also describe disease progression. They have been shown to possess the potential to differentiate between localized and advanced disease, determine Gleason score, cancer metastasis, and biochemical recurrence post-radical prostatectomy. A study by Brase et al. identified miR-141 and miR-375 as the most distinct markers for tumor progression after analysis of sera from individuals with metastatic PCa and localized tumors [188]. The diagnostic and discriminatory potential of miR-21 in urinary samples of PCa patients compared to patients with BPH was identified in a study by Stuopelyte et al. This study also identifies a panel, consisting of miR-21, miR-19a, and miR-19b, having higher diagnostic potential than that of the PSA test [191]. The elevated serum expression in a combination of 4 miRNAs (miR-20a, miR-21, miR-145, and miR-221) was shown to significantly differentiate between low-risk from intermediate/high-risk score patients [192]. Another signature consisting of miR-20a, as well as miR-17, miR-20b, and miR-106a was identified to differentiate between high and low-risk PCa. An elevation in the panel was correlated with advanced tumor stage and shorter time to BCR in PCa patients who have undergone post-radical prostatectomy [193]. In a recent systematic review and systematic reanalysis of public data by Rana et al., two miRNAs (miR-148a-3p and miR-582-5p) were demonstrated to be constantly prognostic of BCR [[194], [195], [196]]. In a study by Suer et al., an analysis of serum from 20 recurrent and 20 non-recurrent PCa patients associated the downregulation of miR-424 and the upregulation of miR-527 with recurrent PCa, emphasizing their prognostic potential [197]. A study by Bidarra et al. analyzed the plasma miRNA content of a cohort of 252 PCa patients and 52 asymptomatic controls. Findings correlate miR-182-5p and miR-375-3p with more advanced pathological stages, with miR-375-3p more significantly predicting metastatic development with 48.72% sensitivity and 75.59% specificity [198].

MiRNA levels have been shown to describe PCa patients' response to therapy. The serum levels of miR-21 in docetaxel chemotherapy-resistant hormone-refractory PCa patients were found to be elevated [199]. Another study by Liu et al., associated an increase in serum miR-200 and a decrease/maintained miR-17 level pre-docetaxel treatment with higher rates of no-response and shorter survival [200]. MiRNA levels detected in peripheral blood mononuclear cells (PBMS) can also be predictive of side effects such as acute genitourinary (AGU) radiotoxicity. A cohort of PCa patients monitored before, during, and a month after RT demonstrated the overexpression of three miRNAs, miR-21, miR-146a, and miR-155, suggesting their radio-sensitivity. MiR-21 levels increase during RT and then significantly decrease a month after in PCa patients who experienced AGU radiotoxicity and those who did not. miR-146a and miR-155 levels also increased during RT, however they did not significantly change a month after RT in those with AGU radiotoxicity. This suggests the use of miR-21 as an indicator of higher apoptosis rates and radiosensitivity while miR-146a and miR-155 could potentially be associated with higher inflammatory responses [201].

Clinical efforts are aiming at evaluating the diagnostic and prognostic powers of miRNAs in PCa patients. Two ongoing clinical trials NCT04662996 and aim to determine the predictive capacity of miRNAs. NCT04662996 is expected to enroll a cohort of 50 castration-resistant PCa patients anticipated to undergo chemotherapy or novel hormonal therapy, and assess miRNA expression in blood samples before and after therapy. While NCT02366494 intends to enroll 60 PCa patients and validate the capacity of 200–300 exosomal miRNAs, identified by RNA sequencing, in predicting the response to ADT. Subsequently, the top-five most prevalent miRNAs will be reported [202,203]. Moreover, an observational study NCT04835454 that anticipates to enroll 120 participants aims to examine the diagnostic role of miR-194 as well as its potential in being a target for cancer treatment [204].

In summary, determining miRNAs that could be used as biomarkers for PCa diagnosis and prognosis is still a necessity and an ongoing work. The latter is instrumental in the therapeutic route of PCa deliberated to each patient (Tables 2 and 3).

**Table 2:** List of miRNAs with diagnostic, prognostic, and therapeutic potential in PCa.

MiRNAs	Specimen	Usage	Refs.
miR-106b	Serum	D	[183]
miR-141-3p	Serum	D, P	[183.^184^]
miR-21	Serum, Urine	D, P, T	[183,185,199]
miR-375	Serum	D	[183]
miR-141	Blood	D, P	[[185], [186], [187], [188]]
miR-98-5p, miR-326, miR-152-3p, miR-326, miR-4289	Plasma	D	[189]
miR-223, miR-24, and miR-375	Serum	D, P	[190]
miR-148a	Urine	D	[191]
miR-375	Urine, serum	D, P	[188]
miR-21, miR-19a, and miR-19b	Urine	D	[191]
miR-20a, miR-21, miR-145, and miR-221	Serum	D, P	[192]
miR-20a, miR-17, miR-20b, and miR-106a	Blood	D, P, T	[193]
miR-148a-3p and	Tissue	D, P	[[194], [195], [196]]
miR-582-5p	Tissue	D, P	[197]
miR-424	Serum	D, P	[197]
miR-527	Serum	D, P	[197]
miR-182-5p, miR-375-3p	Plasma	D, P	[198]
miR-200, miR-17	Serum	D, P, T	[200]
miR-21, miR-146a, miR-155	Plasma	T	[201]

Abbreviations: miRNAs; MicroRNAs, miR; microRNA, D; diagnosis, P; prognosis, T; therapy.

**Table 3:** Various miRNAs-based therapeutic strategies investigated in PCa.

Therapeutic Strategy			MiRNA	Setting	Delivery route	Delivery vector	Effect
MiRNA Inhibition	AMOs	Antagomir	miR-221/222	*In-vivo*	Intratumoral injection	-	Restoring p27/ Inhibiting PC3 tumor cells proliferation [97]
		LNA	MiR-21	*Ex-vivo*	-	-	Restoring PTEN/ Inducing DU145 tumor cells apoptosis [205]
		PNA	MiR-21	*In-vivo*	Intravenous injection	-	Restoring PTEN/ Inducing DU145 tumor cells apoptosis [205]
		-	miR-141/ miR-375	*In-vivo*	Intraperitoneal	PEI nanoparticles	Reducing PC3-derived tumor growth [206]
		-	miR-150/ miR-638	*In-vivo*	Intraperitoneal	PEI nanoparticles	Suppressing MDM4/ Reducing melanoma tumor growth and inhibition of metastasis [206]
	miRNA sponge		miRNA-767-5p	*Ex-vivo*	-	-	Restoring TET1/ Inhibiting human enzalutamide resistant prostate cells proliferation and invasion [207]
MiRNA replacement	miRNA mimics		MiR-185	*Ex-vivo*	-	-	Inhibiting AR and repressing CDC6/ Inhibiting migration, invasion, and tumor formation ability of LNCaP cells [208]
			MiR-34a	*In-vivo*	Tail vein	Chitosan nanoparticles	Suppressing MET and Axl/ Deceasing growth of PC3-derived bone-metastatic PCa and inducing autophagy [209]
			MiR-145	*In-vivo*	Intravenous injection	Polyarginine-conjugated PEI	Decreasing PC3 peritoneal prostate tumor growth and increasing survival time [210]
			MiR-15a and MiR-16-1	*In-vivo*	Intravenous injection	Aptamer-conjugated ATE	Decreased growth and increased survival time in human PCa bone metastasis mice model [211]
			MiR-145	*In-vivo*	Intravenous injection	Mimic conjugated with polyarginine	Radiosensitizing PC3 and LNCaP tumors [212]

Abbreviations: miRNA; microRNA, miR; microRNA, AMOs; anti-miRNA oligonucleotides, LNA; Locked nucleic acid, PNA; peptide nucleic acid, PEI; polymer nanoparticle polyethyleneimine, ATE; Atelocollagen, PTEN; Phosphatase and TENsin homolog, MDM4; MDM4 Regulator of P53, TET1; Tet methylcytosine dioxygenase 1, AR; androgen receptor, CDC6; Cell Division Cycle 6, MET; mesenchymal to epithelial transition, Axl; AXL Receptor Tyrosine Kinase, PCa; prostate cancer.

## MiRNAs as therapeutic agents in PCa

**Fig. 4:**
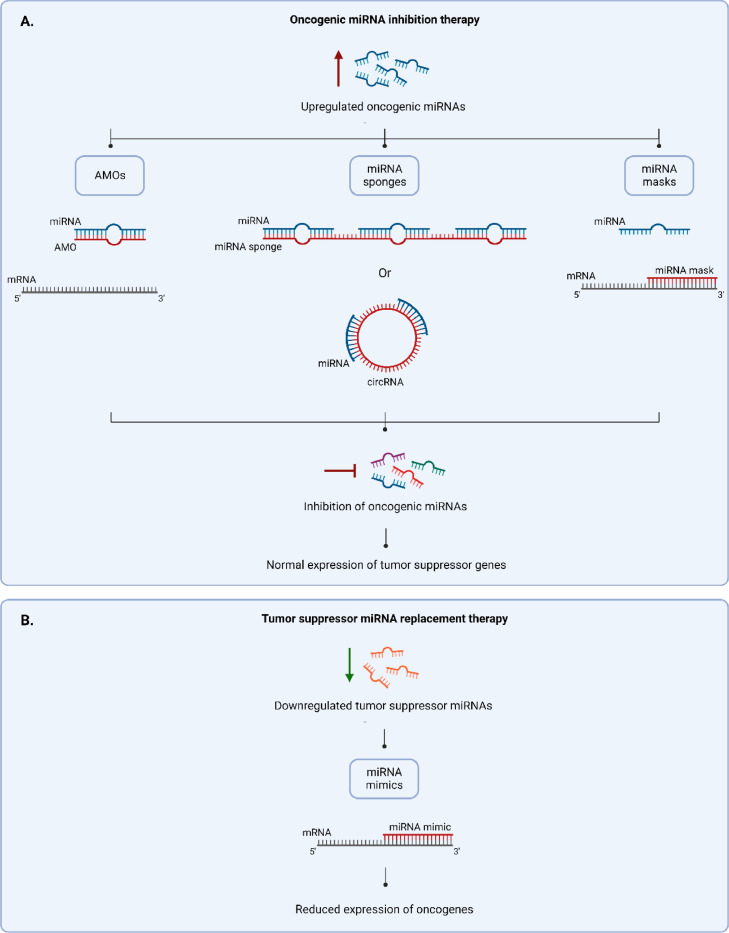
Schematic representation of the potential therapeutic strategies that can be used to treat miRNA dysregulation in PCa. 4A. Strategies used to inhibit upregulated oncogenic miRNA include: AMOs that bind to miRNAs directly, miRNA sponges that have multiple binding sites for miRNAs, and miRNA masks that bind to the mRNA target of miRNA. 4B. One strategy used to restore downregulated tumor suppressor miRNAs is miRNA mimics that act in the same manner as tumor suppressor miRNAs by binding to the mRNAs of oncogenic genes and inhibiting their expression. Abbreviations: miRNA; microRNA, mRNA; messenger RNA, AMOs; anti-miRNA oligonucleotides, circRNA; circular RNA, PCa; Prostate cancer.

MiRNAs can be used not only for diagnosing PCa but also for developing therapeutics against it. MiRNA-based therapeutics aim to reverse the pathological miRNA alterations that enhance the proliferation, invasion, and metastasis of tumors. Since oncogenic miRNAs repress the translation of mRNA of tumor suppressor genes and tumor suppressor miRNAs repress those of oncogenes, inhibiting upregulated oncogenic miRNAs and restoring downregulated tumor suppressor miRNAs can be effective for treating PCa (refer to Fig. 4).

### Oncogenic miRNA inhibition therapy

The loss-of-function modulation of miRNA can be achieved through different methods. The most common one is using anti-miRNA oligonucleotides (AMOs) which are synthetic, about 17–22 nucleotide-long, anti-sense oligonucleotides that are designed to be complementary to their target miRNA especially at the seed region [213,214] (refer to Fig. 4A). They act as miRNA antagonists that anneal to the mature miRNA inhibiting the miRNA-guided action of RISC on the tumor suppressor mRNA resulting in the inhibition of tumor cells proliferation and metastasis [215]. Since their unmodified forms are inefficient in inhibiting miRNAs *in vitro* and *in vivo*, chemical modifications can be introduced mainly in at the C2’ position of the sugar ring and/or in the backbone to increase their potency [213]. One example of chemically modified AMOs used to treat PCa are Antagomirs, designed by Krutzfeldt et al. [216]. These were the first modified AMOs used *in vivo* [216], and they are widely used to inhibit miRNAs *in vivo* in PCa. In these AMOs, the 3’ end is conjugated to cholesterol, thiol moieties replace the non-bridging oxygen of few phosphate groups at the 3’ and 5’ ends, and O-methyl modification on the C2’ of ribose sugar (2’OMe) is applied to the whole strand. For example, the intratumoral injection of anti-miRNA-221 and anti-miRNA-222 antagomirs resulted in the knockdown of the corresponding oncogenic miRNAs and the restoration of tumor suppressor p27, reducing the growth of PC3 cells-derived subcutaneous xenografts in mouse [97]. 2’OMe modification of AMOs is used alone in some studies such as in the one done by Li et al. where using 2’OMe-modified anti-miRNA21 increased the sensitivity of prostate tumor cells to apoptosis and decreased their motility and invasion [217]. Locked nucleic acid (LNA) and peptide nucleic acid (PNA) are also among the modifications applied to AMOs used for targeting PCa. LNA is a modified ribonucleic acid in which the 2’hydroxyl group and 4’carbon of the ribose ring are connected by a methylene bridge which locks it in a C3′-endo conformation and makes it bicyclic [213]. PNA is a ribonucleic acid in which the ribose phosphate is replaced by N-(2-aminoethyl) glycine; these nucleic acids are connected by peptide bonds [213]. In a study conducted by Kim et al., LNA- and PNA- modified anti-miRNA21 strands showed an anti-tumor effect as they inhibited the survival of DU145 PCa cells *in vitro* and reduced tumor proliferation in the mouse subcutaneous tumor xenograft pre-transfected with this inhibitor [205]. In addition, the intravenous injection of PNA-type anti-miRNA21 significantly reduced the metastatic tumors in the mouse bone metastasis model.

Another strategy to silence oncogenic miRNA is using miRNA sponges (refer to Fig. 4A). MiRNA sponges are long RNA transcripts that are stably encoded by an expression vector with a strong promoter [218]. These long nucleic acids sequester miRNAs through tandem complementary target sites hence providing multiple binding sites for the miRNAs to prevent them from binding to their actual tumor suppressor mRNA targets [219]. For example, Jung et al. designed a multipotent miRNA sponge that have multiple perfect and bulged-matched miRNA-binding sites for miRNA-155, miRNA-21, miRNA-221/222 which are overexpressed oncogenic miRNAs in prostate and other types of cancer [220]. This sponge successfully reduced these 4 miRNAs simultaneously in breast and pancreatic cancer cells resulting in sensitizing them to cancer drugs and reducing their migration, and it might have the same effect on PCa cells. Studies have shown the presence of natural endogenous RNAs, long noncoding RNA (lncRNA) and circular RNA (circRNA), which can act in the same way as synthetic sponges [221]. For example, Zhendong Xiang et al. showed that CircRNA-UCK2 has binding sites for miRNA-767-5p and that inhibiting PCa cells proliferation and invasion *ex vivo* can be achieved by expressing this circRNA in PCa cells through lentiviral expression plasmids which leads to the sponging and inhibition of miRNA-767-5p and hence increases the expression of - the target gene of this miRNA- TET1 [207], a tumor suppressor that one of its functions in PCa is inhibiting matrix metalloproteinases - enzymes required in cell invasion and epithelial-mesenchymal transition - by promoting the expression of their inhibitors TIMP2 and TIMP3 (tissue inhibitors of metalloproteinases 2 and 3) [222]. Zhendong Xiang et al. showed that knocking down TET1 in PCa cells reverses the effects of overexpressing CircRNA-UCK2 and restores PCa cells proliferation and invasion which highly suggests that TET1 overexpression is what inhibits cell proliferation and invasion [207].

Several other promising strategies of inhibiting oncogenic miRNAs have been employed for other types of cancers. MiRNA masks are chemically modified antisense oligonucleotides (refer to Fig. 4A). Opposite to an AMO which has a sequence complementary to the miRNA, a miRNA mask has a sequence complementary to the mRNA and similar to the miRNA. Therefore, while an AMO can undergo base pairing with the oncogenic miRNA and inhibit it directly, a miRNA mask can undergo base pairing with the mRNA - of the tumor suppressor gene - that is targeted by the oncogenic miRNA. Hence, by this way, a miRNA mask prevents the access of the oncogenic miRNA-directed RISC to the mRNA of the tumor suppressor gene allowing normal translation of these tumor suppressor proteins [223]. Another strategy is genome editing using CRISPR (clustered regularly interspaced short palindromic repeats)-Cas 9 (CRISPR-associated protein 9). This method can be used can to delete oncogenic miRNA genes or mutate them such that the miRNA precursors cannot be recognized by the miRNA biogenesis machinery, which gives longer lasting effects than transiently transfecting the cells with the miRNA inhibitors [224].

### Tumor suppressor miRNA replacement therapy

MiRNA replacement therapy aims to restore the normal physiological levels of downregulated tumor suppressor miRNAs. The main strategy is using miRNA mimics which are chemically modified synthetic miRNA-like RNA duplexes (refer to Fig. 4B). They simulate the action of endogenous miRNA and can be loaded and processed by RISC to inhibit oncogenic mRNAs [225]. Single-stranded miRNA mimics that simulate the mature guide miRNA can be used, however, these double-stranded mimics are 100–1000 more effective [225]. To further improve their efficacy, modifications such as 2’-O-methylation are applied, yet they should be limited because the mimic needs to be loaded in RISC [226]. Transfecting LNCaP cells with 2’O-Methylated miRNA-185 mimic successfully restored the level of the tumor suppressor miRNA-185 and suppressed their tumor formation ability *in vivo* [208]. MiRNA replacement-based treatments, as well as miRNA inhibition, can be also used to sensitize the tumor cells to tumor. Gong et al. succeeded in sensitizing LNCaP- and PC3- derived tumor xenografts to radiotherapy through treating them with pre-miRNA-145 mimics, and the tumor growth was significantly reduced [212].

MiRNA inhibition and replacement strategies have shown a promising therapeutic potential to treat tumors, yet there are some serious problems that need to be addressed. Unmodified miRNA mimics and inhibitors are prone to rapid degradation by serum nucleases and rapid clearance by renal excretion, which causes a very short systemic circulation time [227]. Moreover, unmodified nucleic acids delivered systemically can activate the innate immune response and induce immunotoxicity [227]. In addition, one of the main concerns is delivering the mimics and inhibitors to the target cancer tissues with effective penetration to avoid any side effects [228]. Some of these issues can be addressed by chemical modifications such as those discussed earlier. PNAs, LNAs, C2’ moieties and other modifications show increased nuclease resistance, reduced immunotoxicity and enhanced binding affinity to their targets [229]. Yet, despite these improved properties, carriers are needed to improve the efficacy of these therapeutics *in vivo*.

### Delivering miRNA therapeutics

Many viral and non-viral delivery systems that enhance the stability, decrease the side effects, and increase the accuracy and cellular uptake of miRNA-based therapeutics have been reviewed [230]. Viral vectors are efficient carriers, however, their immunotoxicity and other side effects limit their applicability [231]. On the other hand, non-viral vectors were considered less efficient, however, with recent advances in nanotechnology, nanoparticles are showing promising characteristics in delivering miRNA inhibition and replacing therapeutics in PCa [231].

Many recent studies show the efficiency of polymeric nanoparticle-based vectors in delivering miRNA therapeutics *in vivo*. One of these is the cationic polymer nanoparticle polyethyleneimine (PEI), yet it exerts cytotoxic effects [232]. Conte et al. synthesized Poly(3-hydroxybutyrate) (PHB) nanoparticles complexed with low molecular PEI [232]. PHB is a biodegradable polymer with no immunotoxicity and is degraded by nonspecific lipases and esterases which makes it a good delivery system for miRNA, however, its negative charge limits its application. Combining PEI with PHB created cationic nanoparticles with very minimal cytotoxicity that protected miRNA-124 mimics from nuclease degradation for extended duration. miRNA-124-complexed nanoparticles showed an enhanced transfection efficiency compared to a commercial transfection agent and impaired PC3 cells tumorigenicity by suppressing the translation of CPT1A oncogene mRNAs. In another study, Zhang et al. reduced the immunotoxicity and increased cell biocompatibility of PEI by the introduction of a disulfide linkage (SSPEI), and they added a cell permeable peptide poly-arginine (R11) as targeting ligand to enable PCa cells-specific interaction only [210]. The resulting R11-SSPEI nanoparticles showed no significant cytotoxicity, increased stability of miRNA-145 mimics, enhanced cellular specificity and uptake. After systemic delivery of SSPEI/miRNA-145 complex via intravenous injections, the prostate tumor showed high uptake value in prostate tumor but low uptake in the major clearance organs, and the complex inhibited the peritoneal prostate tumor growth. This R11 peptide was used also to deliver the pre-miRNA145 mimics in the study by GONG et al. previously described [212]. Kunz et al. also used PEI nanoparticles to deliver LNA and 2’-OMe modified AMOs to successfully inhibit tumor proliferation and metastasis in *in-vivo* prostate carcinoma and melanoma mouse models [206].

In addition to PEI, Chitosan is a natural biodegradable polysaccharide with no immunogenicity and toxicity that can easily complex with miRNA due to its cationic character [233]. Systemic delivery of tumor suppressor miRNA-34a encapsulated in chitosan polymeric nanoparticles induced apoptosis and inhibited the growth of subcutaneous and bone metastatic PCa mouse models [209].

Lipid nanoparticle-based vectors are used to deliver miRNA-based therapeutics too. Among these are cationic niosomes which are biodegrabale vectors made up of non-ionic surfactants for improved stability, neutral lipids for improved cellular uptake, and cationic lipids for charging the nucleic acid [234]. Ghaffari et al. delivered miRNA-15a and miRNA-16–1 mimics through cationic niosomes conjugated with polyethylene glycol (PEG) to PC3 cells which induced their apoptosis by downregulating the Bcl-2 [235]. Moreover, other biomaterials like Atelocollagen (ATE) have been reported as delivery systems. In a study done by Hao et al., delivering miRNA-15a and miRNA-16-1 mimics using ATE conjugated with RNA aptamer that targets prostate-specific membrane antigen (PSMA) expressed by some PCa types inhibited the growth of bone-metastatic prostate tumor mouse model [211].

Despite the advancements in miRNA-based therapeutics and their delivery strategies, further preclinical studies are needed to verify the efficacy and safety of these methods. Currently, there are no miRNA treatments being tested clinically for PCa [236].

## Conclusion

It is well established that the expression of some miRNAs is dysregulated in diverse types of cancer including PCa. Several mechanisms may lead to an aberrant profile of miRNAs that may range from chromosomal modifications, altered epigenetic regulations, and abnormal miRNA biogenesis. As such, prostate cells with dysregulated miRNA expression may have a shifted balance between tumor-suppressing and tumor-promoting/oncogenic miRNAs. Consequently, crucial cellular processes may be affected such as AR signaling, proliferation, apoptosis, EMT, and invasion; all leading to PCa development and progression.

Interestingly, the dysregulated profiles of miRNAs form signatures that have been exploited to determine specific biomarkers for early diagnosis, prognosis, and prediction of treatment efficacy against several cancer types together with PCa. The latter is unique in the sense that blood, urine, and semen may be used as sources for biomarkers detection. This is of particular interest for clinical use since miRNAs are present in these samples which may be extracted non-invasively and repeatedly from the patients. To detect and determine specific miRNA biomarkers, different high-throughput tools evolved and were employed over the years. Mainly, qRT-PCR, microarray, NGS, and nCounter-based methods are being used. Each technique involves advantages and pitfalls whereby employing one over the other depends on many factors such as the research question/targets, sample types, and resources. To date, an ideal miRNA high-throughput detection technique that combines the following characteristics does not yet exist. In summary, this method should have high sensitivity to detect low levels of miRNAs, high specificity to confidently discriminate one nucleotide difference between the targets, the ability to provide quantitative outputs of miRNAs expression levels, steps that are easy/simple to perform, and equipment/reagents that are readily available [1]. Taking the lack of a best suitable detection method into consideration, along with the fact that PCa studies were carried out on a limited number of patients, in addition to the inconsistency of data obtained between the different studies, few published work presented one particular miRNA described clearly as a biomarker for PCa. Thus, more efforts are required to profile and validate miRNA candidate biomarkers in large cohort studies.

Despite all advances, management of mCRPC still presents challenges emphasizing the need for novel treatment and drug delivery approaches. miRNA-based therapeutics have been considered as potential strategies to target PCa. However, till now, no miRNA treatments have been clinically tested for PCa, as miRNA-based therapeutic approaches are still naïve lacking further preclinical studies to validate their efficacy and safety.

## Availability of data and materials

Not applicable.

## Financial support and sponsorship

None.

## Ethical approval and consent to participate

Not applicable.

## Consent for publication

Not applicable.

## Copyright

© The Author(s).

## CRediT authorship contribution statement

**Fatima Ghamlouche:** Conceptualization, Project administration, Supervision, Investigation, Methodology, Writing – original draft, Writing – review & editing, Visualization, Validation. **Amani Yehya:** Conceptualization, Project administration, Supervision, Investigation, Methodology, Writing – original draft, Writing – review & editing, Visualization, Validation. **Yousef Zeid:** Methodology, Writing – original draft, Validation. **Hiam Fakhereddine:** Methodology, Writing – original draft, Validation. **Jhonny Fawaz:** Methodology, Writing – original draft, Validation. **Yen-Nien Liu:** Conceptualization, Writing – review & editing, Visualization, Validation. **Mohamed Al-Sayegh:** Conceptualization, Writing – review & editing, Visualization, Validation. **Wassim Abou-Kheir:** Conceptualization, Project administration, Supervision, Writing – review & editing, Visualization, Validation.

## Declaration of Competing Interest

All authors declared that there are no conflicts of interest.
